# Spatial Niche Partitioning in Sub-Tropical Solitary Ungulates: Four-Horned Antelope and Barking Deer in Nepal

**DOI:** 10.1371/journal.pone.0117917

**Published:** 2015-02-25

**Authors:** Krishna Prasad Pokharel, Tobias Ludwig, Ilse Storch

**Affiliations:** Wildlife Ecology and Management, Faculty of Environment and Natural Resources, University of Freiburg, Freiburg, Germany; University of Fribourg, SWITZERLAND

## Abstract

Differential resource use allows a diversity of species to co-exist in a particular area by specializing in individual ecological niches. Four-horned antelope *Tetracerus quadricornis* is endemic to the Indian subcontinent and has a restricted distribution in Nepal and India; however, the barking deer *Muntiacus vaginalis* is relatively common throughout its wide distribution range. We wanted a better understanding of their habitats and how these two similarly sized solitary ungulates manage to coexist in lowland Nepal. We used fecal pellet belt transect surveys in the Babai valley, Bardia National Park to study the habitat associations of both species. We found empirical evidence that four-horned antelope prefer hill sal forest and deciduous hill forest at higher elevations, whereas barking deer preferred riverine and sal forest in lower elevations. We found a clear niche differentiation of four-horned antelope and barking deer that made the coexistence of these similarly sized solitary ungulates possible. Hence, resource partitioning is the key to coexistence of these solitary ungulates, and the fine-grained habitat mosaic of different forest types in the study landscape appears to be the underlying feature. Therefore, maintaining the habitat mosaic and preserving valuable hill sal and deciduous hill forests will facilitate the coexistence of herbivores in sub-tropical regions.

## Introduction

Niche theory posits that species partition environmental space along environmental gradients [1] as well as in geographical space [2,3]. An ecological niche also describes the biotic and abiotic resources affecting the fitness, i.e., successful reproduction of an individual or a population of a species. Morphologically similar and phylogenetically close sympatric species are expected to have high niche overlap and competitive interactions under conditions of limited resources [4]. Furthermore, the realized niche of herbivores is in part determined by predation [4]. Hence, potentially competing sympatric herbivores are expected to develop niche differentiation to avoid or lessen competition [4–7]. The mechanism allowing for niche differentiation occurs mostly along the three niche axes: spatial, temporal, and trophic [8]. The spatial aspect of the niche (habitat) is the major niche factor most frequently partitioned [4,9]. With sympatric herbivores in sub-tropical regions, differential use of resources in space as well as the spatial nature of habitat mosaics presumably facilitates coexistence. Therefore, studies on species-habitat relationships and niche differentiation of sympatric animals are important to understand coexistence mechanisms and to support biodiversity conservation.

The four-horned antelope *Tetracerus quadricornis* de Blainville, 1816, (FHA) is a vulnerable species on IUCN Redlist and is endemic to Nepal and India [10]. In the national Redlist of Nepal, FHA is listed as *data deficient* [11]. Scattered populations of this species are distributed from the Himalayan foothills to peninsular India. Furthermore, reliable estimates of the global population of this species are not available and its habitat use is poorly known [12]. Unlike FHA, barking deer *Muntiacus vaginalis* Boddaert, 1785, (BD) are widespread throughout South- and Southeast Asia with a global conservation status as *least concerned species* on the IUCN Redlist [13]. However, in Nepal, BD is listed as *vulnerable* [11]. FHA and BD both are solitary creatures with similar morphology (shoulder height 55–65cm, adult weight 18–21kg) [14–18]. FHA has a narrow distribution range in the lowlands [19–21] whereas BD are widely distributed from lowland to the high mountains [13]. FHA have been described as inhabiting relatively open and dry deciduous forest in hilly terrain [18,19,21–23] whereas BD use a variety of habitats from dense forest [14,24,25] to scrub grassland and thorny shrub land [26]. Populations of these species are decreasing mainly because of habitat loss and fragmentation, and illegal hunting [10,11,13]

To date most reports on the habitat associations of FHA and BD are qualitative descriptions and a quantitative assessment of their habitat preferences is still lacking. Little is known about the habitat requirements particularly of the FHA in Nepal, and about habitat partitioning between the two species. To fill this gap, we studied the habitat use of sympatric FHA and BD in lowland Nepal and modelled the results. Modelling of species distributions has two main goals: explanation of species habitat requirements and spatial prediction [27]. In the last decade, great advances in different techniques have been made to achieve these goals [28]. Whereas spatial prediction is the main purpose in many studies, our sole research interest was in assessing those habitat variables that explained the occurrence for FHA and BD. To achieve this goal, we used two different but complementing modelling approaches: boosted regression trees [29,30] and generalized linear modelling [31]. Furthermore, based on predictions made by niche theory, that ‘coexisting species should differ in their ecological requirements by at least some minimal amount to avoid competitive exclusion’ [6]; we discuss the partitioning of the major habitat factors of these sympatric species. We believe that our results will aid in understanding the coexistence mechanism of these similarly sized solitary ungulates, and inform conservation management of these threatened species throughout the area of their distribution.

### Methods

Ethics statement

We used an indirect sign survey (non-invasive) method and were conscious not to disturb animals in the field while walking along the transect. We neither collected any kind of animal samples nor performed any kind of laboratory-based works. We are thankful to the Department of National Parks and Wildlife Conservation (DNPWC) for the research permission.

Study area

**Fig 1:**
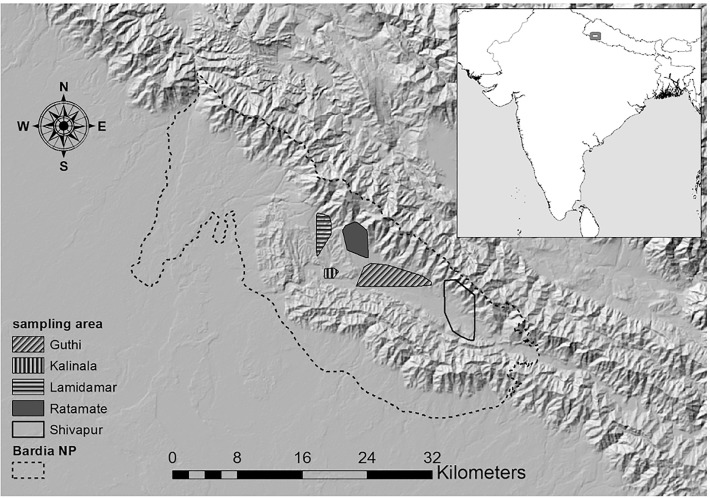
Survey subareas within the Babai valley, Bardia National Park, Nepal. Their delineation encompasses the outermost sampling points. While Lamidamar subarea was sampled only in 2012, Ratamate and Shivapur were sampled both in 2012 and 2010. Further validation data from 2010 was sampled in Guthi and Kalinala subarea. Map courtesy: background hillshade calculated based on USGS [62] and park boundary source: Department of National Parks and Wildlife Conservation, Government of Nepal.

This study was conducted at Babai valley in the southeastern part of Bardia National Park (28°23′0″N, 81°30′0″E). The park is located in lowland Nepal near the Indian border, 390km west of Kathmandu (Fig. 1). It is the largest national park in the lowland Terai covering an area of 968 km^2^. The Babai valley lies in the Churia range, also called the Siwalik Hills. It is a river valley extending from Parewaodar to Chepang. The park has a subtropical monsoonal climate with three distinct seasons; monsoon, winter, and dry (http://www.dnpwc.gov.np assessed on 19 March 2014). Monthly mean temperature in the area ranges from a minimum of 10°C in January to a maximum of 45°C in June. Most of the rain (1560–2230 mm) occurs during the monsoon season from June to September (source: Department of Hydrology and Meteorology, Nepal: 2004 to 2009). From October until early June the weather is dry, the days are warm, and the nights are cool [32].

The vegetation association within the study area is sub-tropical, consisting of a mosaic of floodplain communities with riverine forest along and near the Babai river and its tributaries, and with large areas of climax sal *Shorea robusta* forest on adjoining lowland areas. There are patches of grassland, locally known as *phatas*, scattered in the sal forest. Phatas are dominated by tall and perennial grasses, such as kans grass *Saccharum spontaneum* and blady grass *Imperata cylindrica*, and are actively managed by the park management to support the spotted deer *Axis axis* population as a main prey for tiger *Panthera tigris*. Most of the lower range of the Siwalik hills are dominated by sal forest, hereafter hill sal forest. Upper elevations are covered by dry deciduous forest with shorter trees, hereafter deciduous hill forest, which is dominated by axle wood *Anogeissus latifolia* and by *Terminalia tomentosa*. There are a few patches of mixed successional forest in nearby areas of tributaries of the Babai river, which consists of *Acacia catechu, T. tomentosa* and dense stands of *Phoenix humilis*.

Data collection

Pilot study.

Dense vegetation in most of the areas made direct observation of study animals difficult. Therefore, we used a pellet-count method. Unlike sambar *Rusa unicolor*, Kerr, 1972, and chital *Axis axis* Erxleben, 1777, FHA and BD use latrines [33,34], i.e., they repeatedly deposit pellets at a point location. Additionally, sambar and chital pellets are generally much larger than those of FHA and BD. To evaluate our observations, based on suspected latrines of the study animals, we installed motion-sensor cameras (stealth cam STC-1550, model number D-40, USA). We placed the cameras at about 40cm height. In case of completely burnt grasses, we hid the camera with tree twigs so that the animals would not be distracted by it. We checked each camera every 2–3 days for 15 days. The latrine with additional fresh pellets where FHA or BD was photographed defecating was considered as a dung-pile of that respective species. The study animals did not visit the same latrine every day. Therefore, we assumed that individuals of FHA and BD both use multiple latrines and those latrines are randomly located throughout the used habitat.

**Fig 2:**
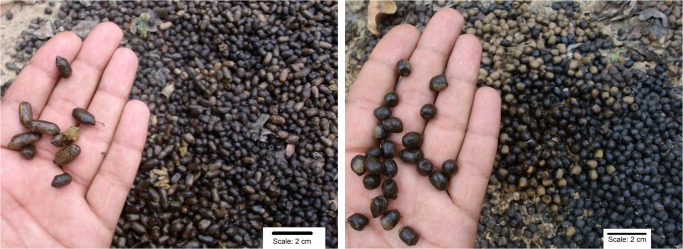
Images showing typical structure of faecal pellets of FHA (left) and BD (right) with latrine of respective animals. Scale bar on the lower right of each photograph refers to the foreground (the hand).

Usually latrines of FHA were bigger in size, sometimes up to 1.5m in diameter, whereas those of BD did not exceed 1m in diameter. In addition to the size of a latrine, there was considerable variation exhibited in the pellet shape for both species. Unlike the typical comma-shaped pellets of BD—similar to the observation of Dinerstein [15], FHA pellets were elongated. In some cases the shape of pellets of both animals was cylindrical and pointed at one end. In such a case, the visual ratio of length to diameter of FHA pellets was bigger than that of BD (Fig. 2). In some latrines, very small pellets of young ones were also observed. The colour of pellets also varied from light grey to black. White pellets were due to the presence of higher soil content in the diet. In such a case, FHA pellets were bigger in diameter than those with a normal diet, while BD pellets were much longer and thinner than usual. We concluded that it was possible to distinguish both species from their dung piles.

Field survey.

We derived information on habitat use of both species by counting dung piles along transects of fixed width. Transects were oriented north to south to include almost all the available vegetation types [35]. The survey covered three different areas within the Babai valley, viz., the Shivapura, Ratamate and Lamidamar areas (Fig. 1). We used belt transects of 20m width, which were placed in the field with a random first start. Four people walked parallel to each other at a distance of 5m apart and at an average speed of 0.5 to 0.7km per hour, depending on the topography and vegetation types, to ensure high detection probability. The gap between adjacent transects was 150m whereas the length of transect varied from 200m to 4km depending on the topography and site accessibility. We used a 20m radius sampling plot to assess the different habitat variables along transects (Table 1). When we encountered the latrines, we assessed habitat variables as *used habitat* with latrines at the centre. We assessed *available habitat* at regular intervals of 150m long transects.

A total transect length of about 130km was traversed in the study area from February to May 2012. Of 1993 sampling plots, we found 461 to be used by FHA and 920 by BD. The mixed forest and grassland types were not represented by enough data points. We therefore reduced the dataset to the four forest types (Table 1) resulting in 1904 sampling plots with 457 FHA and 872 BD as used habitat, respectively.

**Table 1:** Predictors of study species occurrences measured in the field (they can be divided into the following categories: Topography, Forest stand, and Ground Layer).

Variable	Code	Measure/explanation
*Topography*		
Elevation	Elev.m	Elevation above sea level (m) at plot centre
Slope	slop.d	Steepness of slope (in 1°steps) at plot centre
*Forest stand*		
Forest type	for.typ	Terai sal forest (1), Riverine forest (2), Hill sal forest (3), Deciduous hill forest (4),
Tree height	Tre.ht	Average height of all trees > 3m
Canopy cover	Canopy	Average canopy cover of all trees (%)
*Ground Layer*		
Ground cover	Grnd.cvr	Ground cover (%)
Grass	Grss.cv	Proportion of grass cover out of total ground cover (%)
Grass Height	Grss.ht	Average grass height (cm)
Shrubs/regeneration	Shrb.cv	Proportion of shrubs and regeneration out of total ground cover (%)
Shrubs/regeneration height	Shrb.ht	Average shrubs/regeneration height (< 3m)

Statistical analysis

We used two different modelling techniques to explore the relationships between habitat and the occurrences of FHA and BD. Since little is known about FHA and BD in lowland Nepal, we first deployed boosted regression trees (BRT) to rank variables according to their influence on the study species. BRT analyses produce partial dependence plots, which provide powerful basis for interpretation of results [30]. We checked these plots for squared relationships of our variables with study species occurrence. Second, we used generalized linear models (GLM) to compute fitted functions and confidence intervals to further assess the credibility of our explanations.

Boosted Regression Trees (BRT).

Boosted Regression Trees analysis is an advanced machine learning technique. It uses boosting, a flexible method that combines hundreds of regression trees to reveal complex patterns in the data and thus is resistant to over-fitting. The process automatically fits polynomial functions and model complex interactions between predictor variables [30]. The important parameters of BRT are learning rate and tree complexity. The lower the learning rate (lr), the higher will be the number of trees required to reach the minimum error of the model. Tree complexity (tc), the number of nodes in a tree, determines the depth of interactions. Therefore, a smaller lr and a higher tc are preferable [30]. Because the number of trees should exceed 1000 [30], we used a tc of 5 and a lr of 0.002 with Bernoulli distribution (discrete distribution with 2 possible outcomes) for our data set. After an initial model with the full set of variables we performed a test of deviance change under variable reduction and built a second model with the reduced set of variables for each species.

Generalized Linear Models (GLM).

GLMs allow for various response and error term distributions in the model [31]. We used logistic regression with a logit-link function for parameter estimation to create response curves for FHA and BD along environmental gradients. To avoid collinearity [36], we considered r_s_< 0.7 to be an acceptable correlation between predictor variables. Only covariates grass- and shrub cover were highly negatively correlated. We decided to omit the variable grass cover since it ranked lower in BRT-models for both species (see S1 Table). Of nine remaining covariates we built an *a priori* full model using single-term variables and quadratic terms according to visual interpretation of BRT effect plots and our own expectations. For model selection, we used an information-theoretic approach [37] based on Akaike’s Information Criterion (AIC). The AIC-IT approach compares different models that are based on a-priori formulated hypotheses about the focal species [38]. We built all possible variable combinations and ranked our models by the change in Akaike’s Information Criterion (ΔAIC). Finally we performed model averaging in order to calculate a relative importance for each variable for comparison with BRT output.

Model validation.

The BRT procedure automatically outputs the results of a tenfold cross validation. We extracted the explained deviance and area under curve (AUC) and compared them with the corresponding values from a fivefold cross validation for our generalized linear models. The AUC-value gives the probability that, from a randomly chosen presence/absence pair, the observed presence obtains a higher predicted probability of occurrence [39]. Therefore, AUC provides a measure of discrimination ability, which is acceptable with values > 0.7 [40]. However, since our focus was more on explanation of species-environment relationships, we also assessed model performance by checking calibration curves. These curves plot the observed prevalence over bins of the predicted values, thus showing how well the predictions fit the real data. The slope of a calibration curve expresses model reliability with the diagonal (slope = 1) representing perfect calibration. Refinement, i.e., the range of predictions along the x-axis, is also referred to as sharpness [41] and is another important measure of model performance in addition to discrimination and calibration. Two external validation sets were available: one with 958 samples from mapping FHA in the Kalinala region of Bardia National park between April and May 2010, and a small one with 89 samples from the Chure hills, south of Sauraha in Chitwan National park, Nepal, mapped between April and May 2011. We used these data sets to further evaluate the performance and transferability of our final FHA model by extracting AUC and plotting calibration curves.

For all statistical analysis, we used the open source statistical software R version 3.0.0 (R Development Core Team 2013), with the packages ‘gbm’ version 2.1 [42], ‘MuMIn’ version 1.10.0 [43], and ‘PresenceAbsence’ version 1.1.9 [44].

## Results

### Model performance

Simplified BRT models explained 18.2% of the deviance in the full dataset for FHA and 14.0% of the deviance for BD. Based on results of cross validation, the explained deviances are 12.2% for FHA and 9.4% for BD, whereas the better performance of the FHA model is also reflected by its higher measure of discrimination ability (AUC = 0.74 for FHA compared to 0.69 for BD). Fivefold cross validation of the FHA model built with logistic regression yielded the same performance measures as those from BRT (Table 2), thus reflecting low uncertainty from choice of the modelling approach. For BD, the deviance explained with logistic regression was lower than that explained with BRT, which automatically includes interactions. The external validation of the FHA model, which was based on independent data from Bardia National Park, revealed good credibility for the species-habitat relationships found. The explained deviance with 15% was even higher than that for the calibration data and discrimination ability was the same as derived from internal cross validation of the original model (Table 2). When we applied our FHA model to the smaller dataset from another National Park, we were able to explain 22% of the deviance and discrimination ability with AUC = 0.74 was as good as those with the datasets from Bardia National Park. Hence, our model could be applied not only to other regions in Bardia National Park but even to other areas along the Nepalese Terai.

**Table 2:** Performance measures based on cross validation for boosted regression trees (BRT) and generalized linear models (GLM) and application to independent data (only FHA).

	Cross validation(BRT/ GLM)		Independent data (Kalinala/ Chitwan)	
species	D^2^	AUC	D^2^	AUC
FHA	12.2%/ 12.2%	0.74/0.74	14.9%/22.4%	0.73/0.74
BD	9.4%/ 7.7%	0.69/ 0.69	-	-

D^2^: explained predictive deviance

AUC: area under the receiver operating curve (ROC)

### Variables explaining FHA and BD occurrence

Topographical variables and forest type contributed to almost three quarters of the explained deviance in BRT models for both species. The reduced BRT models retained four of the ten initial variables. While that for FHA kept the order of relative variable importance, the reduced BRT model for BD experienced a shift with shrub cover ranking slightly higher than forest type (S1 Table). Our procedure of GLM averaging resulted in eight models with ΔAIC<2 for FHA and nine such models for BD. Relative influences were highest for elevation, forest type, and slope, thus confirming our results from BRT. However, in our averaged GLMs for BD all other variables had much lower relative influences than in GLMs for FHA.

**Fig 3:**
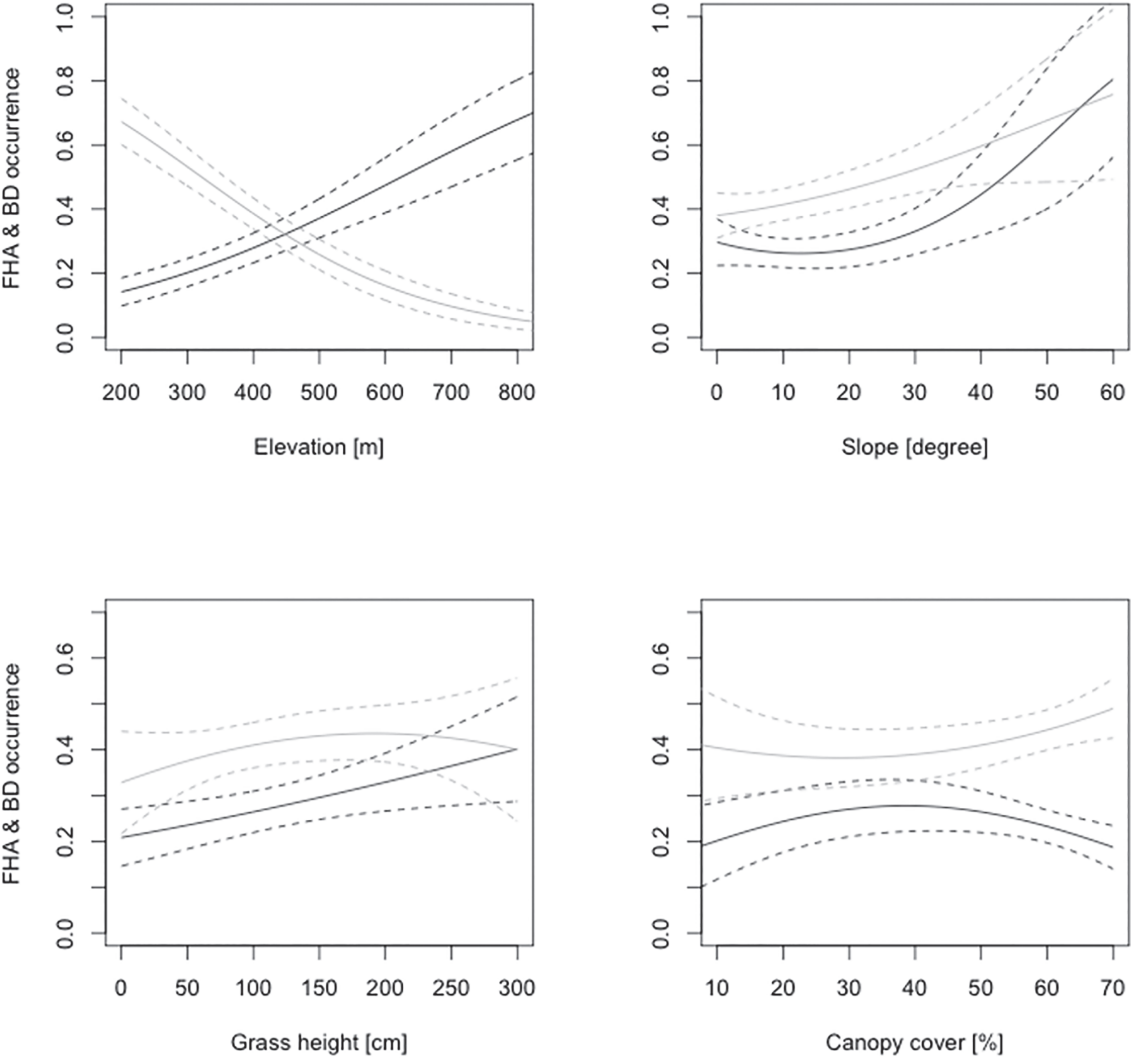
Predicted probability of occurrence for FHA (black lines) and BD (grey lines), during the dry season, in Bardia National Park, with respect to topographic and forest structure variables (95% CI). Occurrence probability was allowed to vary with the variable under consideration, while other covariates were held constant at their median values.

**Fig 4:**
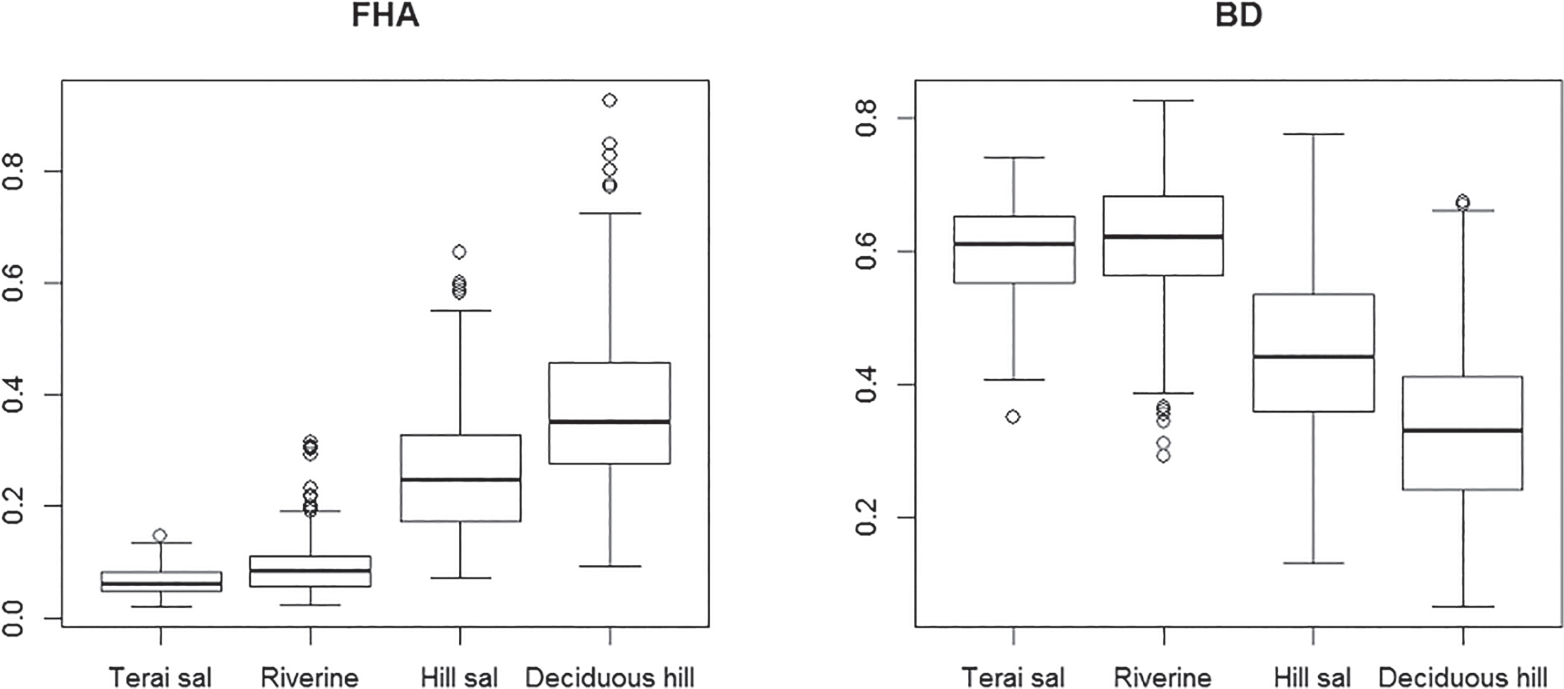
Predicted probability of occurrence for FHA (left) and BD (right) for different forest types during the dry season in Bardia National Park. Occurrence probability was calculated based on the original dataset.

Elevation as one of the most influential explanatory variables had opposing effects on FHA and BD, respectively (Table 3). While both species had about the same probability of occurrence between 400 and 500 metres, partial responses indicate BD occurrence probability to increase towards lower elevations, while FHA was found mainly towards higher altitudes (Fig. 3). Although both species occurred in all four forest types, we also found that FHA occurrence was higher in hill sal and deciduous hill forests, while BD exhibited the strongest responses to riverine and Terai sal forest (Fig. 4). Further, FHA seemed to respond positively to steeper slopes and higher grass stands (Table 3, S1 Fig.). In contrast, BD´s responses to slope and grass height were much less pronounced and results suggest a curvilinear relationship of BD with grass height. Canopy closure was of almost equal importance for FHA and BD but our study species showed differential responses to this variable. FHA´s probability of occurrence was highest at intermediate values between 30 and 40%, BD´s occurrence probability steadily increased until the highest observed crown closure of about 70 percent. Our BRT model suggested shrub cover to be of equal relative importance for the occurrence of BD as forest type. This was no longer supported by our GLM (S1 Table). However, BD was positively associated with shrub cover while FHA did not seem to respond to this variable at all. The same pattern was observed with ground cover. However, this time BD was the species which did not respond whereas FHA exhibited a negative relationship to this variable.

**Table 3:** Averaged model parameters (Estimate), their standard errors (SE) and error probabilities (p) for four-horned antelope (FHA) and barking deer (BD).

	FHA			BD		
	Estimate	SE	p	Estimate	SE	p
(Intercept)	-4.27E+00	1.14E+00	0.0002	1.83E+00	4.50E-01	0.0000
Canopy	3.12E-02	2.34E-02	0.1825	-5.19E-03	1.67E-02	0.7557
I(Canopy^2)	-3.89E-04	2.67E-04	0.1462	1.65E-04	1.44E-04	0.2532
Elev.m	6.82E-03	4.03E-03	0.0909	-5.78E-03	6.54E-04	0.0000
I(Elev.m^2)	-4.17E-06	5.53E-06	0.4513	-	-	-
for.typ Riverine	-1.37E-01	3.51E-01	0.6971	3.23E-01	1.92E-01	0.0923
for.typ Hill sal	9.88E-01	3.35E-01	0.0032	-3.60E-01	1.85E-01	0.0517
for.typ Deciduous hill	8.92E-01	3.63E-01	0.0141	-4.58E-01	2.24E-01	0.0406
Grs.ht	3.58E-03	1.27E-03	0.0050	1.88E-03	2.66E-03	0.4794
I(Grs.ht^2)	-	-	-	-2.82E-06	1.00E-05	0.7785
slop.d	-2.40E-02	2.16E-02	0.2672	2.04E-02	5.87E-03	0.0005
I(slop.d^2)	8.07E-04	4.83E-04	0.0951	-	-	-
Tre.ht	-3.39E-02	1.60E-02	0.0343	-1.23E-02	1.23E-02	0.3161
Grnd.cvr	-3.49E-03	3.21E-03	0.2777	1.85E-03	2.43E-03	0.4463
Shrub.cv	-8.04E-04	3.50E-03	0.8182	2.97E-03	2.66E-03	0.2643

## Discussion

This is the first study showing that four-horned antelope (FHA) use hill sal and deciduous hill forest in lowland Nepal. Furthermore, dung pile sampling revealed a clear niche differentiation of FHA and barking deer (BD) along the altitudinal gradients, which makes the coexistence of these similarly sized solitary ungulates possible in lowland Nepal. A particular issue of critical importance here is whether the latrines placed in the habitat could reliably reflect the study animal’s preference for various habitat and/or habitat factors. Pellet-count techniques have been widely used to map the habitat used by a number of mammals [45–47]. But some authors [48] have questioned on reliability of the technique. However, pellet counts have revealed the similar results of relative habitat use by FHA as obtained from direct observation [19,49]. Furthermore, our findings on preference of BD for riverine and Terai sal forest is similar to the findings of Storaas and Wegge (unpublished data cited In [17]) who used radio-telemetry. Therefore, use of pellet-count technique to assess the used habitat of FHA and BD can be taken as a reliable method.

### Model reliability

**Fig 5:**
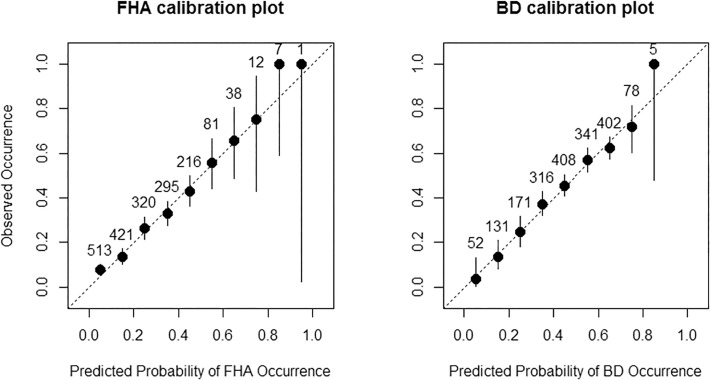
Calibration plots of generalized linear models for FHA (left) and BD (right). Observed occurrences as proportion of sites surveyed are close to the ideal slope represented by the dotted diagonal. Vertical lines represent confidence intervals for a binomial distribution. Figures above the points give the number of cases in each bin.

**Fig 6:**
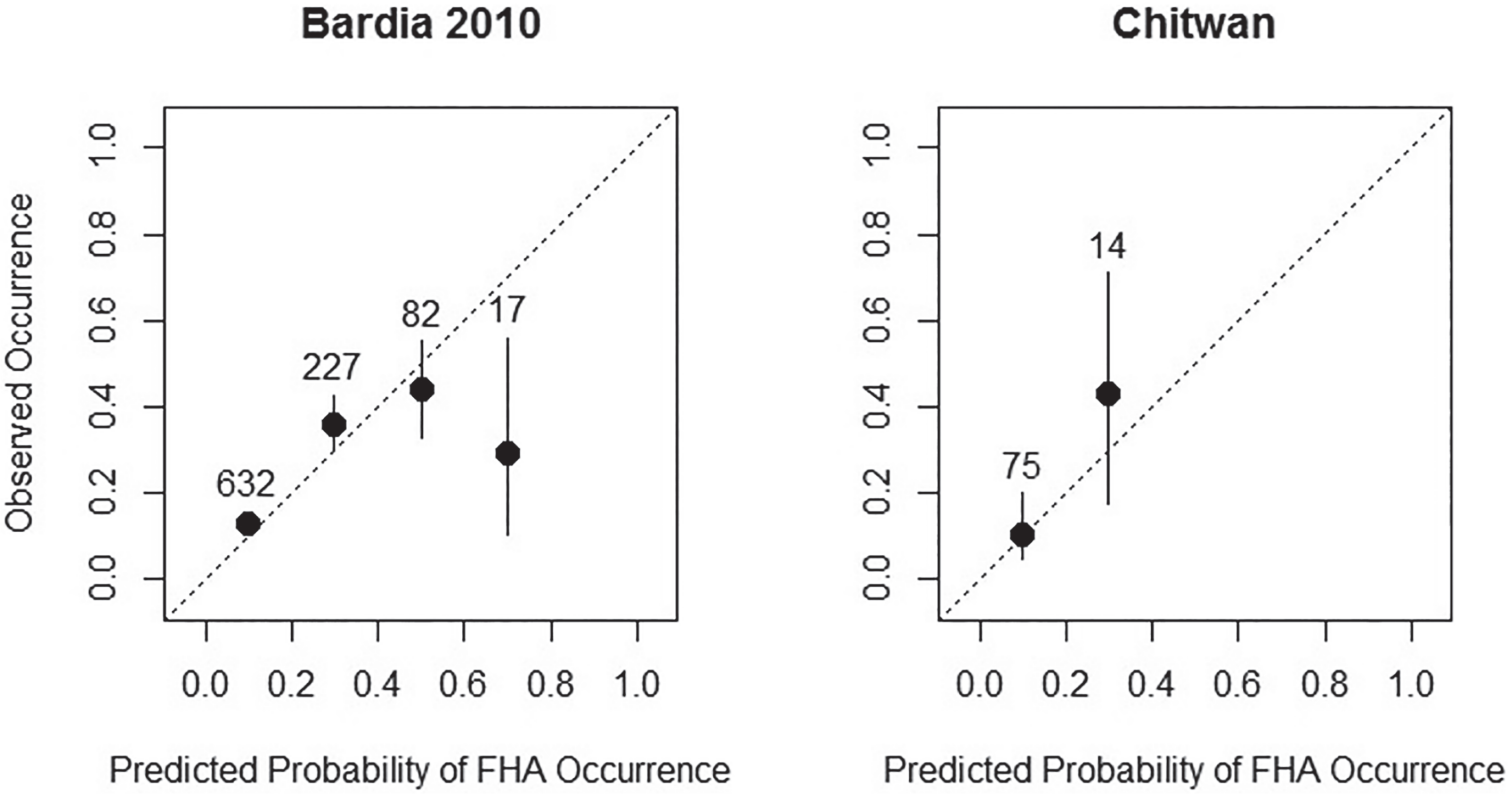
Calibration plot of final GLM for FHA applied to independent validation data from Bardia NP (2010) and from Chitwan NP, middle Nepal. Vertical lines represent confidence intervals for a binomial distribution. Figures above the points give the number of cases in each bin.

Our models explain the occurrences of FHA and BD in Bardia National park with topography and forest structure variables during the end of winter and the onset of the dry season. Models for FHA attained a better fit and a higher predictive power than those for BD. This reflects the general pattern that distribution models for rare species with smaller ranges gain higher accuracy than those for species with large ranges [27] because FHA is only locally distributed and BD is common throughout the Indian subcontinent and south-east Asia. In turn, FHA seems to be the habitat specialist and BD a generalist. Nevertheless, our cross-validated models for BD attained almost acceptable discrimination ability with AUC = 0.69 and most importantly, yielded a good calibration and refinement (Fig. 5). We were also able to apply our FHA model to independent data from Bardia National Park and to Chitwan National Park, ~ 200km away from this studies sampling areas. These external validations confirmed the cross validation results with high discrimination abilities of AUC = 0.74 (Table 2) although fit along the calibration diagonal was less pronounced and the range of predicted probabilities was smaller (Fig. 6) than with the calibration data set. Nevertheless, results showed that our calibrated models are suitable to locally predict FHA and BD in different regions of the Terai in Nepal.

### Habitat characteristics of four-horned antelope and barking deer

In this study we presented topographical and forest structural variables that best explained the occurrence of FHA and BD in lowland Nepal between February and April. Our results suggested that during this transition of winter to dry season, FHA was confined to higher elevations in Bardia NP while BD appeared to prefer lower elevations. Elevation however did not directly affect the species’ distribution. Rather, elevation is correlated with other more functionally relevant predictors such as temperature, rainfall, and solar radiation [50] that lead to the change in habitat features and its quality to support the occurrence of species. In Babai valley, there is a noticeable change in vegetation composition and other habitat variables such as availability of water and air moisture level with altitude. Those variables in turn determine the occurrences of other wildlife, threats from predators, and frequency of wild fires during the dry season as well. Since the study area is a river valley, lower elevations have very good water sources, whereas at higher elevations, water sources are localized in gorges, so the moisture level decreases remarkably with increasing elevation. Consequently, forests at lower elevations, i.e., Terai sal forest and Riverine forest, contain taller trees and exhibit higher canopy and shrub cover. Severity of wildfire is higher in hill sal/ deciduous hill forests than in riverine and Terai sal forest but can be high on grasslands in the valleys. Therefore, the vegetation composition at higher elevation is characterized by fire tolerant species with low breast height diameter and stunted height. Although, the dominant tree species in hill sal forest is sal *Shorea robusta* trees, these are not as tall and dense as they are in Terai sal forest. Furthermore, deciduous hill forest, which replaces the hill sal forest in higher elevations, has the highest grass cover on the forest floor. Deciduous hill forest is relatively drier and more open than Terai sal forest and riverine forest which have taller trees with denser canopy and relatively higher level of moisture all year. Ahrestani et al. [51] found that the occurrence of FHA was negatively associated with plant available moisture (PAM) while BD responded positively to this measure. In our study, we found a positive relationship between occurrence of FHA and forest types’ hill sal- and deciduous hill forest, which is comparable to the findings of Ahrestani et al. [51]. In fact, the occurrence probability of FHA increased with smaller trees, higher grass cover, and intermediate crown cover. Krishna et al. [19] also found FHA occurrence to increase with lower tree height and tree-savanna habitat. On the contrary, BD exhibited a negative relationship with these savanna-like forest types in our models. The reason behind this appears to be the availability of moist and dense forest at lower elevations. Hence, FHA and BD seem to exhibit a niche differentiation in lowland Nepal along an altitudinal gradient, which is manifested by the use of different forest types.

In Babai valley, we found both species in almost all vegetation types. However, FHA had a much higher preference for the hill sal forest and deciduous hill forest but avoided all other forest types including grasslands (Fig. 4). At the same time, BD though exhibited a more generalist pattern of habitat use with higher preference for the riverine forest, Terai sal forest and mixed forest, which are clearly avoided by FHA. Our findings of a preference for hilly areas by FHA are similar to the findings of Khan et al. [52] in Gir, India where FHA inhabits hilly areas. Preference for deciduous forest by this species are compatible with the findings of Krishna et al. [19] in Bandipur National Park, India. On the other hand, our findings of a preference for riverine forest and sal forest by BD are in complete agreement with the findings of Dinerstein [53] in Bardia National Park. Our findings of preference for closed forest by BD are similar to the findings of Brodie and Brockelman [24] in KhaoYai National Park in Thailand; and Pokharel and Chalise [25] in the mid-hill region of Nepal. However, our results are contradictory to the findings of preference for scrub grassland and thorny shrubland in Hainan Island, China by Teng et al. [26]. Such differential use of habitat in different parts of BD distribution range might be the results of evolutionary adaptations to local predators and other environmental variables. Furthermore, use of different forest types might also be the cause of different anti-predatory behaviour of the study species. As far as anti-predatory behaviour is concerned, FHA depends largely on making itself inconspicuous; in the presence of predators it freezes, lies down and freezes, or runs to cover and freezes [22] while BD escape into the dense forest [14]. Therefore, FHA might have used hill sal forest and deciduous hill forest more where there was neither dense canopy as in Terai sal forest and riverine forest nor openness as in grasslands. This finding supports the theory that differential preference for the available forest types is the main reason of coexistence of these solitary ungulates in landscape.

It is likely that these species respond differently in other seasons. This is supported by the application of our FHA calibration model to the validation data set, which was sampled between March and May 2010. Interestingly, the effects of forest type, grass height, tree height, and canopy cover were very similar to those found with our calibration data (Fig. 5). However, the effects of topography (slope, elevation) were much less pronounced. One explanation is that the sampling period for the validation data had been much drier than that of our calibration data, hence forcing FHA to find its resources at lower elevations where it still preferred the savanna-like forest types. BD almost always remains in the forest during the hot-dry season [53] showing its ‘shade loving’ behaviour. It is reasonable that BD preferred those forest types where moisture level was relatively higher, due to denser canopy with taller trees compared to other forest types.

The role of competition and predation for this differentiation is less clear. Forests at lower elevation are the major habitat of other wildlife. The most common herbivore is the chital (spotted deer) with densities of up to 50 individuals/km^2^ [54]. It uses grassland as well as adjacent forest types such as Terai sal forest, riverine forest, and mixed forest [15,55]. Dense forests also are the habitat of sambar *Rusa unicolor* and wild boar *Sus scrofa*, which attract predators like tiger *Panthera tigris*, and leopard *Panthera pardus*. Using higher elevation, FHA might have selected the areas with low level of interspecific competition and reduced threats from major predators. In this regards, BD seems to be more tolerant to interspecific disturbance than FHA, leading to the observed differential habitat use and niche partitioning. In an evolutionary context, FHA might have been an inferior competitor compared to BD but was able to populate the drier and less productive forest types.

The possible explanation for using steeper areas by both species may be due to differential predator pressure in the Bardia National Park. Main predators of these species are tiger *Panthera tigris* and leopard *Panthera pardus* [54,56,57]. Many lines of research indicated that spotted deer *Axis axis*, which prefer grasslands and riverine forest [55], are the primary prey for tiger and leopard [56,58]. As the availability of suitable prey and hunting success are the most important factors determining the habitat use by carnivores [59], grasslands and associated sal forest and riverine forests are the attractive areas for those predators. Natural predators may have an important role for the species coexistence when each species has its own specialist predator. Those natural enemies maintain the density of each species independently [60]. Therefore, the occurrence of tiger and leopard might also have impacted the space use pattern by the study species. In addition to the reduced availability of preferred prey, steeper areas might be unsuitable for predators to catch the prey with reasonable effort. Furthermore, poaching of wildlife for various purposes such as bush meat, and trade has been an ongoing process for centuries, particularly in tropical regions [61]. FHA and BD are game species that have been poached in Bardia National Park. Mostly, these animals are hunted for subsistence [11]. Moreover, most of the security posts are in the valley regions with increased human disturbance. Therefore, it is reasonable that both FHA and BD use the steeper areas to minimize the risk of predation and avoid the human disturbance.

## Conclusion

Our study provides empirical evidence that four-horned antelope prefer hill sal forest and deciduous hill forest at higher elevations in lowland Nepal. These findings increase our scarce knowledge about the ecology of this rare ungulate species and are directly relevant for conservation. Furthermore, we found a clear niche differentiation of four-horned antelope and barking deer that made the coexistence of these similarly sized solitary ungulates possible in lowland Nepal. Hence, resource partitioning is the key to coexistence of these ungulates, and the fine-grained habitat mosaic of different forest types in the study landscape might be the underlying feature. FHA can be seen as a habitat specialist and BD a generalist, which is reflected in higher model accuracy for FHA. These findings have to be verified at larger spatial scale extents to predict distribution ranges for both species in Nepal. In Bardia National Park and other parts of the Terai in Nepal, conservation authorities would profit by maintaining the habitat mosaic and preserving valuable hill sal and deciduous hill forests. This will facilitate the coexistence of herbivores in lowland Nepal and especially ensure the survival of inferior competitors and specialists like FHA.

## Supporting Information

S1 Fig Partial dependency plots showing the marginal effects of the four most relevant predictor variables of reduced BRT models for the four-horned antelope (left) and barking deer (right) occurrence. Higher fitted function values denote positive species responses and vice versa.
(TIFF)

S1 Table Influence of predictor variables that could explain the occurrence of FHA and BD in percentage of explained deviance. “Full” shows influences of all variables, “reduced” influences of variables whose removal would result in significant increase in unexplained deviance. For the variables’ explanation, see Table 1.
(DOCX)
