# Supplementary figures and images for: Spatial Niche Partitioning in Sub-Tropical Solitary Ungulates: Four-Horned Antelope and Barking Deer in Nepal
Source: PLoS One. 2015 Feb 25;10(2):e0117917. doi: 10.1371/journal.pone.0117917 (PMC4340944; doi:10.1371/journal.pone.0117917)

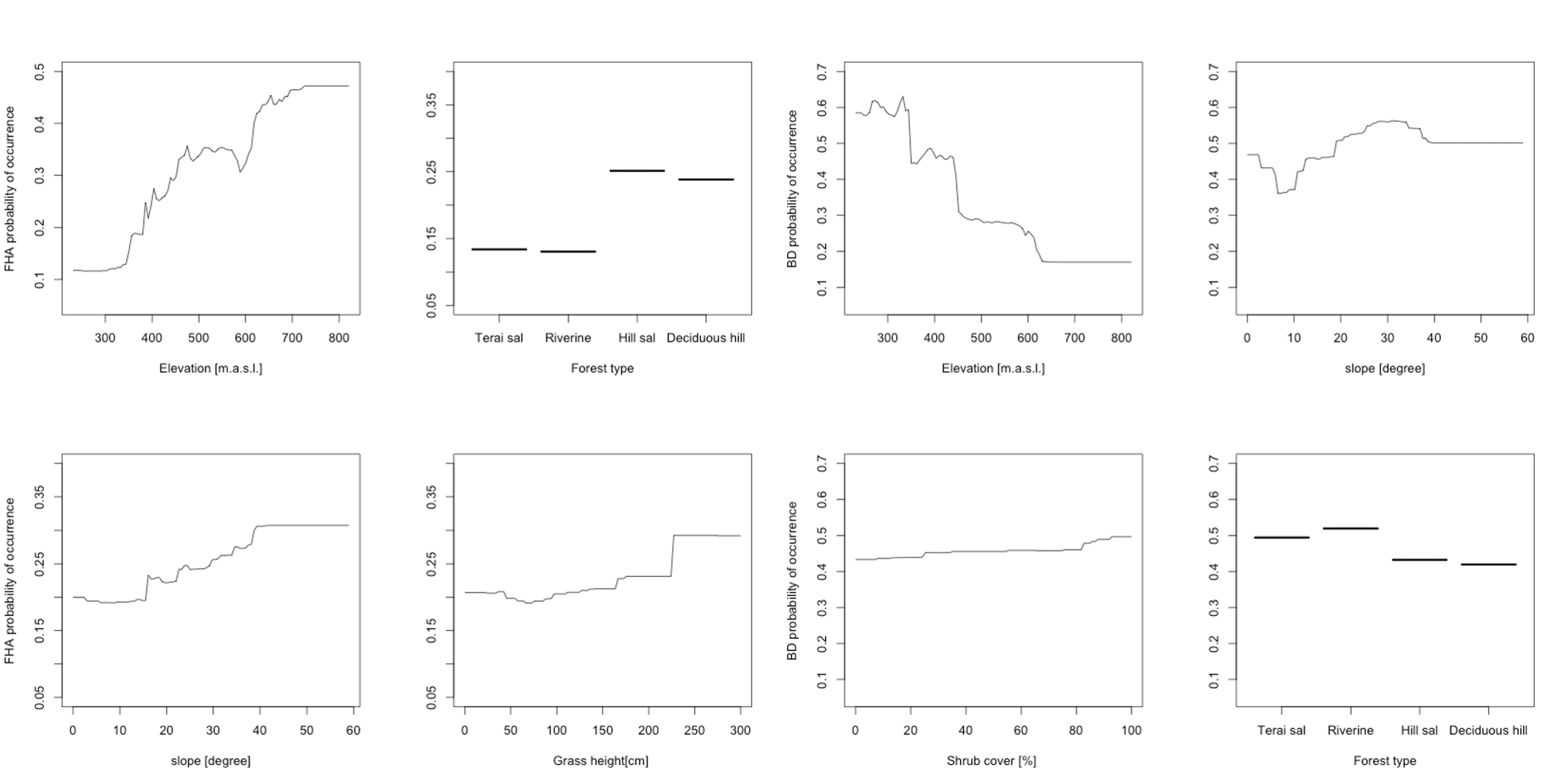

Supplement: S1 Fig — Higher fitted function values denote positive species responses and vice versa. (TIFF) [file pone.0117917.s001.tiff]
