# Supplementary material for: Spatial Niche Partitioning in Sub-Tropical Solitary Ungulates: Four-Horned Antelope and Barking Deer in Nepal
Source: PLoS One. 2015 Feb 25;10(2):e0117917. doi: 10.1371/journal.pone.0117917 (PMC4340944; doi:10.1371/journal.pone.0117917)
Supplement: S1 Table — “Full” shows influences of all variables, “reduced” influences of variables whose removal would result in significant increase in unexplained deviance. For the variables’ explanation, see Table 1. (DOCX) [file pone.0117917.s002.docx]

Table S1.

| Variables | | Full | Full cumulative | Reduced | RI GLM |
| --- | --- | --- | --- | --- | --- |
| Four horned antelope | |  |  |  |  |
| 1 | Elev.m | 47.7 | 47.7 | 57.4 | 0.92/ 0.49 |
| 2 | For.typ | 12.9 | 60.7 | 16.2 | 1 |
| 3 | slop.d | 12.6 | 73.3 | 13.6 | 0.57/0.93 |
| 4 | Grs.ht | 9.2 | 82.5 | 12.8 | 0.98 |
| 5 | Canopy | 4.0 | 86.4 |  | 0.63/0.73 |
| 6 | Tre.ht | 3.4 | 89.9 |  | 0.79 |
| 7 | Shrub.cv | 3.4 | 93.3 |  | 0.27 |
| 8 | Grnd.cvr | 2.9 | 96.2 |  | 0.4 |
| 9 | Grss.cv | 2.0 | 98.1 |  | - |
| 10 | Shrb.ht | 1.9 | 100.0 |  | - |
| Barking Deer | |  |  |  |  |
| 1 | Elev.m | 47.8 | 47.8 | 60.3 | 1 |
| 2 | slop.d | 17.8 | 65.6 | 21.0 | 0.98/0.29 |
| 3 | For.typ | 7.9 | 73.5 | 9.2 | 1 |
| 4 | Grs.ht | 4.3 | 77.8 |  | 0.36/ 0.31 |
| 5 | Canopy | 4.3 | 82.2 |  | 0.49/0.71 |
| 6 | Tre.ht | 4.1 | 86.2 |  | 0.37 |
| 7 | Shrub.cv | 3.8 | 90.0 | 9.5 | 0.42 |
| 8 | Grss.cv | 3.6 | 93.6 |  | - |
| 9 | Grnd.cvr | 3.3 | 96.9 |  | 0.32 |
| 10 | Shrb.ht | 3.1 | 100.0 |  | - |
